# Clinical utility and cost modelling of the *phi* test to triage referrals into image-based diagnostic services for suspected prostate cancer: the PRIM (Phi to RefIne Mri) study

**DOI:** 10.1186/s12916-020-01548-3

**Published:** 2020-04-17

**Authors:** Lois Kim, Nicholas Boxall, Anne George, Keith Burling, Pete Acher, Jonathan Aning, Stuart McCracken, Toby Page, Vincent J. Gnanapragasam

**Affiliations:** 1 Department of Public Health and Primary Care, University of Cambridge, Cambridge, UK; 2 Department of Urology, Cambridge University Hospitals Trust, Cambridge, UK; 3 Urological Malignancies Programme CRUK & Cambridge Urology Translational Research and Clinical Trials Office, University of Cambridge Box 193, Cambridge Biomedical Campus Cambridge CB20QQ, Cambridge, UK; 4 NIHR Cambridge Biomedical Research Centre, Core Biochemical Assay Laboratory, University of Cambridge, Cambridge, UK; 5 Department of Urology, Southend Hospital, Essex, UK; 6 Department of Urology, North Bristol NHS Trust, Bristol, UK; 7 Department of Urology, South Tyneside and Sunderland NHS Trust, Sunderland, UK; 8 Department of Urology, Newcastle Hospitals NHS Trust, Newcastle upon Tyne, UK; 9 Academic Urology Group, Department of Surgery, University of Cambridge, Cambridge, UK

**Keywords:** Prostate cancer, Prostate health index (*phi*), mpMRI, Biopsy, Cambridge prognostic groups

## Abstract

**Background:**

The clinical pathway to detect and diagnose prostate cancer has been revolutionised by the use of multiparametric MRI (mpMRI pre-biopsy). mpMRI however remains a resource-intensive test and is highly operator dependent with variable effectiveness with regard to its negative predictive value. Here we tested the use of the *phi* assay in standard clinical practice to pre-select men at the highest risk of harbouring significant cancer and hence refine the use of mpMRI and biopsies.

**Methods:**

A prospective five-centre study recruited men being investigated through an mpMRI-based prostate cancer diagnostic pathway. Test statistics for PSA, PSA density (PSAd) and *phi* were assessed for detecting significant cancers using 2 definitions: ≥ Grade Group (GG2) and ≥ Cambridge Prognostic Groups (CPG) 3. Cost modelling and decision curve analysis (DCA) was simultaneously performed.

**Results:**

A total of 545 men were recruited and studied with a median age, PSA and *phi* of 66 years, 8.0 ng/ml and 44 respectively. Overall, ≥ GG2 and ≥ CPG3 cancer detection rates were 64% (349/545), 47% (256/545) and 32% (174/545) respectively. There was no difference across centres for patient demographics or cancer detection rates. The overall area under the curve (AUC) for predicting ≥ GG2 cancers was 0.70 for PSA and 0.82 for *phi*. AUCs for ≥ CPG3 cancers were 0.81 and 0.87 for PSA and *phi* respectively. AUC values for *phi* did not differ between centres suggesting reliability of the test in different diagnostic settings. Pre-referral *phi* cut-offs between 20 and 30 had NPVs of 0.85–0.90 for ≥ GG2 cancers and 0.94–1.0 for ≥ CPG3 cancers. A strategy of mpMRI in all and biopsy only positive lesions reduced unnecessary biopsies by 35% but missed 9% of ≥ GG2 and 5% of ≥ CPG3 cancers. Using PH ≥ 30 to rule out referrals missed 8% and 5% of ≥ GG2 and ≥ CPG3 cancers (and reduced unnecessary biopsies by 40%). This was achieved however with 25% fewer mpMRI. Pathways incorporating PSAd missed fewer cancers but necessitated more unnecessary biopsies. The *phi* strategy had the lowest mean costs with DCA demonstrating net clinical benefit over a range of thresholds.

**Conclusion:**

*phi* as a triaging test may be an effective way to reduce mpMRI and biopsies without compromising detection of significant prostate cancers.

**Electronic supplementary material:**

**Supplementary information** accompanies this paper at (10.1186/s12916-020-01548-3).

## Background

Prostate cancer is the most common male cancer in the western world and its incidence is rising. One of the biggest conundrums facing health care systems is how best to detect and diagnose potentially life-limiting disease while not simultaneously over-investigating and finding indolent tumours. The use of multiparametric magnetic resonance imaging (mpMRI) pre-biopsy is now firmly embedded in the prostate cancer diagnostic pathway as a method to try and address this [1–5]. This enables targeting of positive lesions and in the case of negative imaging, avoiding biopsy all together in a proportion of referrals [5]. There remains, however, significant limitation with mpMRI particularly around costs, access, quality control, sustainability and meeting the needs of a growing population of ageing men, only some of whom will harbour lethal disease [6–8]. mpMRI also has significant operator-dependent variability and its negative predictive value is known to vary from study to study depending on scanner type, sequences selected and radiologist experience [9, 10]. As a result, the European Association of Urology (EAU), amongst other bodies, has called for research into pre-MRI triage tests to refine and improve the use of mpMRI [11]. A few studies have now tested combinations of biomarkers and imaging in controlled trials [12–15]. On-line risk calculators are also available and have recently been updated to include mpMRI data in their predictive algorithms [16]. To date, however, few have considered how biomarker-imaging combinations might work to refine the use of tests and hence cost-effectiveness.

The prostate health index (*phi*) is one of the most robustly studied prostate cancer biomarkers in the literature and also one of the lowest cost tests available [17–19]. It has consistently shown better predictive value compared to PSA in detecting prostate cancer in numerous studies in many countries and has been combined with on-line risk calculators [20]. Previous work in our group was the first to combine *phi* with mpMRI demonstrating its utility in triaging who needs re-biopsy from an initial negative investigation [21]. Here we have extended that work to test the use of the *phi* in a multicentre first referral population. Our principle question was if the *phi* test could reduce mpMRI/biopsy use without compromising detection of prognostically important cancers. Importantly, if this could be done in a routine diagnostic service real-world context without controlling for imaging and biopsy methodology.

## Methods

### Patients and data acquisition

Five UK centres took part with each recruiting consecutive men referred from primary care for elevated prostate-specific antigen (PSA) (January 2018 to June 2019.) mpMRI method and reading and biopsies were carried out according to local protocols in order to ground the study in real-world practices. The only stipulation was image-guided targets + systematic biopsies (cognitive or image fusion). Men were excluded if they had (i) a previous biopsy, (ii) pelvic metalwork interfering with mpMRI quality or no mpMRI and (iii) if no biopsy was done after mpMRI. PSA and *phi* assay was taken before biopsy and PSA density (PSAd) calculated using mpMRI-defined prostate volumes. Only men with intact information on key data points, PSA (ng/mL), *phi*, mpMRI, prostate volume, biopsy performed, histological Grade Group and clinical tumour stage (T-stage) were included in the final analysis. Men with missing data from the original 554 were not included in the analysis leaving a final study cohort of 545 (Additional File Fig. S0). Additional File Table S1 details the imaging and biopsy strategy in each unit. Men with mpMRI-negative lesions had systematic sectoral biopsies only. The study was conducted under ethics REC 03/018.

#### *phi* assay

*phi* assays were handled according to the manufacturer’s recommendations (Beckman Coulter). Blood was taken prior to biopsies and before prostate manipulation. Samples were centrifuged and frozen at – 80 °C within 3 h before dispatch to a central laboratory and performed on a Beckman Coulter Access Autoanalyser. Quality Assurance samples were analysed before and after each batch to ensure the validity of the results. All QC results were within Beckman Coulter’s target ranges. *phi* results were not viewed or analysed till after all men had been recruited. There were no adverse events from the extra sampling.

#### mpMRI

mpMRI on 1.5 T or 3 T systems with multi-channel surface phased array coils were performed including standard anatomical and functional imaging (diffusion-weighted and contrast enhanced). Image acquisition and processing was performed in accordance with local standard clinical protocols. In all centres, sequences were evaluated and scored using a Likert scale of cancer probability, based on the Prostate Imaging Reporting and Data ver. 2 (PI-RADS version 2). Prostate volumes were calculated from mpMRI images. Likert 1–2 (M1-M2) lesions were considered mpMRI negative for this study and positive lesions graded as M3-M5. Lesion calling was left to local expertise without specification of reader experience.

### Statistical analysis and decision modelling

The primary end-points were two definitions of prognostically important cancers (i.e. cancers that may shorten life-expectancy if not found). For this, we used International Society of Urological Pathology histological Grade Group 2 or more on biopsy (≥ GG2) and prognostic group 3 or more using the composite Cambridge Prognostic Group (CPG) prognostic score (≥ CPG3) we have previously reported and validated [22]. CPG is a five-tiered multi-factor (PSA, Grade and Stage) prognostic model for non-metastatic prostate cancer shown to have superior discrimination in predicting prostate cancer deaths compared to any other tiered stratification systems [23]. Disease ≥ CPG3 is similar to unfavourable intermediate risk and high-risk disease in the AUA, NICE and EAU systems [24]. Areas under the receiver-operating curve (AUC) and diagnostic test statistics for different strategies were compared to PSA alone: *phi* and PSAd. We adopted a decision modelling approach to bring together information about sensitivity, specificity and costs. A range of potential clinical pathways were modelled and compared *Strategy 1*: mpMRI and biopsy all, *Strategy 2*: mpMRI all and biopsy if positive, *Strategy 3*: mpMRI all and biopsy if PSAd ≥ 0.15, *Strategy 4*: mpMRI all and biopsy if PSAd ≥ 0.1, *Strategy 5*: *phi* all and mpMRI and biopsy if *phi* ≥ 25, *Strategy 6*: *phi* all and mpMRI and biopsy if *phi* ≥ 30. The proportions of men with positive test results in the study cohort were used to calculate cancers detected or missed under each pathway. Additional Figure S2 shows the pathway and cohort percentages corresponding to Strategy 6. Costs for assays, scans and biopsies are given in Supplement Table S2; one-way sensitivity analyses were used to explore the impact of assumptions regarding the cost and risk of sepsis following biopsy. Key outcomes relating to correctly identified cancers (true positives), incorrectly identified cancers (false positives), missed cancers (false negatives), numbers of MRI scans, numbers of biopsies and total costs were then calculated for a hypothetical cohort of 1000 referred individuals.

### Decision curve analysis

Decision curve analysis (DCA) was done to estimate the clinical net benefit outcome, which accounts for the perceived value weighting between the harms of biopsy in those without cancer and the harms of not identifying those with cancer [25]. The value weighting represents the decision-makers’ beliefs about the benefit to harm ratio between cancer detection and unnecessary biopsy. It can be thought of as the risk of cancer at which there is equipoise about whether to proceed to biopsy (risk threshold, RT) and takes values between 0 and 1. A low risk threshold implies that the perceived harm of biopsy is low compared to the benefits of identifying cancer; conversely, a high-risk threshold occurs when biopsy harm is high compared to the benefits of identifying cancers. The clinical net benefit is then calculated (at a given value of RT) as the number of cancers detected minus the number of unnecessary biopsies weighted by RT. Since the perceived point of biopsy equipoise is uncertain, results are presented over a range of RT values. The preferred detection strategy is then indicated by the one with the highest net benefit, at a given RT. The cost per net cancer detected under each strategy is also presented.

## Results

### Cohort description and between centre comparison

The final study population included 545 men with a median age of 66 years, PSA of 8 ng/ml and *phi* of 44 (Table 1). Overall mpMRI results were reported as positive (M3-M5) in 420/545 (77%) with M4-M5 lesions found in 316/545 (58%) (Table 1). The median number of cores taken was 16 for systematic biopsies and 2 for targets (if positive on an mpMRI). Overall, prostate cancer was detected in 349/545 men (64%) and ≥ GG2 cancers in 256/545 (47%). Using the composite CPG score, disease ≥ CPG3 was found in 174/545 men (32%). Additional File Table S1 details the MRI positivity and method of biopsy acquisition for each centre. There were no significant differences between centres in any of these parameters despite no pre-specified standardisation for diagnostic method and reporting [26, 27].

**Table 1:** Descriptive characteristics of the primary study cohort. MRI positive data is shown as the PI-RADS score of ≥ 3 or ≥ 4. Detection rates for cancer are shown for any cancer, and using definitions of ≥ Grade Group 2 (GG2 or ≥ Cambridge Prognostic Group 3 [CPG3]. Excludes 9 men with missing data not included in the analysis

**Cohort descriptors**	***N*** **= 545**
**Age (median) (inter-quartile range)**	66 (60,70) years
**PSA (median) (inter-quartile range)**	8 (6,13) ng/ml
**PHI (median) (inter-quartile range)**	44 (30,69)
**MRI (3–5)**	77%
**MRI (4–5)**	58%
**Any cancer detection**	64%
**≥ GG2 cancer detection**	47%
**≥ CPG3 cancer detection**	32%

### Performance of PSA, PSAd and *phi* in predicting prostate cancer at biopsy

**Fig. 1:**
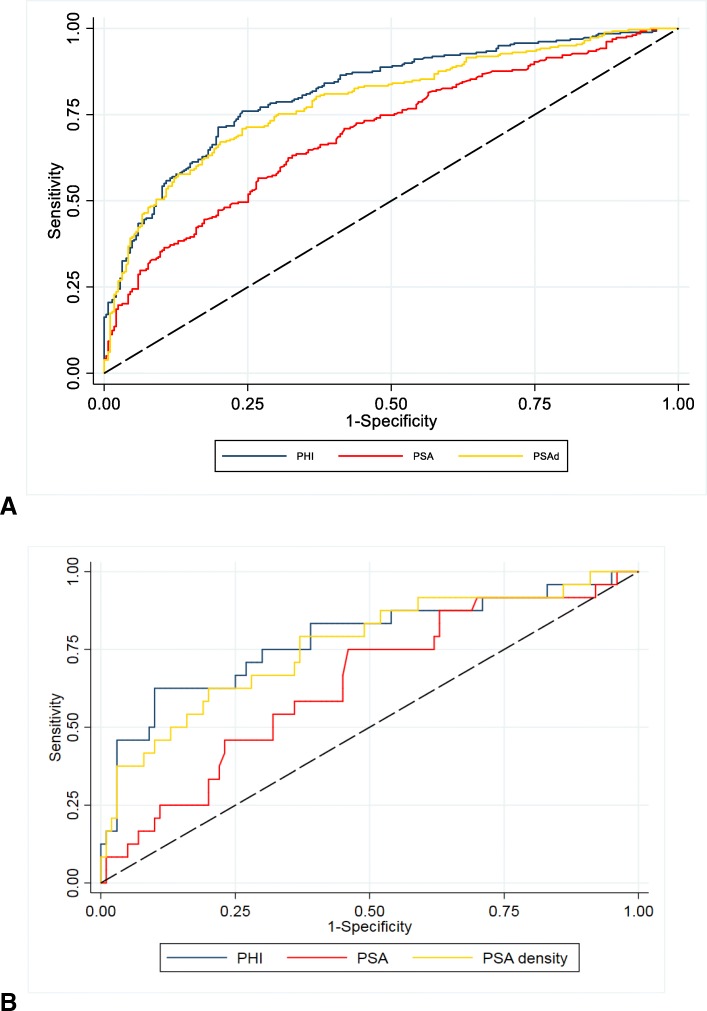
ROC curve illustrating performance of *phi*, PSA, PSAD and mpMRI in predicting cancer diagnosis of ≥ Grade Group 2 (GG2) in the **a** whole cohort and **b** mpMRI-negative men (PI-RADS ≤ 3)

For detection of significant cancers defined as ≥ GG2, the AUCs were 0.70, 0.79 and 0.82 for PSA, PSAd and *phi* respectively (Fig. 1a and Table 2). Both PSAd and *phi* performed significantly better than PSA alone in predicting ≥ GG2 disease (*p* < 0.001) (Table 2). The predictive value of *phi* for ≥ GG2 was also similar across all 5 centres (*p* = 0.67). The combination of PSA, PSAd with imaging increased the AUC to 0.76 and 0.81 respectively (Table 2). Using ≥ CPG3 as an endpoint, the AUCs were 0.81, 0.84 and 0.87 for PSA, PSAd and *phi* respectively (Additional File Figure S1A and Table 2). However, only *phi* was significantly better than PSA in predicting ≥ CPG3 disease (*p* < 0.001) (Table 2). Amongst mpMRI-negative men (*n* = 125), the AUCs for detection of ≥ GG2 cancers were 0.64, 0.76 and 0.78 respectively with both PSAd and *phi* performing better than PSA. Neither PSAd nor *phi* however performed better than PSA in predicting the presence of ≥ CPG3 disease (Fig. 1b, Table 2 and Additional File Figure S1B).

**Table 2:** Descriptive characteristics of the primary study cohort. MRI positive data is shown as the PI-RADS score of ≥ 3 or ≥ 4. Detection rates for cancer are shown for any cancer, and using definitions of ≥ Grade Group 2 (GG2 or ≥ Cambridge Prognostic Group 3 [CPG3]

	**≥ GG2 cancer** **detection**	***p*value** **(vs. PSA)**	**≥ CPG3 cancer** **detection**	***p*value** **(vs.PSA)**
**Whole cohort**				
**PSA**	0.70 (0.66–0.74)	–	0.81 (0.78–0.85)	–
**PSAd**	0.79 (0.75–0.83)	< 0.001	0.84 (0.80–0.87)	0.12
***phi***	0.82 (0.78–0.85)	< 0.001	0.87 (0.84–0.90)	< 0.001
**MRI**	0.63 (0.59–0.66)	< 0.001*	0.63 (0.60–0.66)	< 0.001*
**MRI + PSA**	0.76 (0.72–0.80)	< 0.001	0.85 (0.81–0.88)	0.03
**MRI + PSAd**	0.81 (0.77–0.84)	< 0.001	0.85 (0.81–0.88)	0.08
**MRI +** ***phi***	0.81 (0.78–0.85)	< 0.001	0.86 (0.83–0.90)	0.02
**MRI negative**				
**PSA**	0.64 (0.52–0.76)	–	0.86 (0.76–0.97)	–
**PSAd**	0.76 (0.64–0.87)	0.01	0.95 (0.91–0.99)	0.08
***phi***	0.78 (0.66–0.90)	0.01	0.89 (0.74–1.00)	0.76

### *phi* thresholds in selecting men for referral to mpMRI and biopsy

We next tested different threshold of the *phi* to test its ability to triage men for initial mpMRI and biopsy. Using detection of ≥ GG2 as an endpoint, *phi* cut-offs ≥ 20 and ≥ 30 to refer for mpMRI and biopsy had an NPV of 0.85 and 0.90 respectively and missed 1.1 and 7.7% of tumours (Table 3). With ≥ CPG3 as a detection target, *phi* performance showed even better results with NPVs of 0.94 and 1.0 respectively and with 0% and 4.5% of cancers missed. Of note, using a *phi* ≥ 35 threshold (which we reported for re-biopsy men) produced rather poor sensitivity [21] (Table 3). This suggests that useful *phi* thresholds may be different depending on the detection context. As a comparator, we also analysed the performance of PSAd (noting that this is not usually available before mpMRI). PSAd thresholds of ≥ 0.1, ≥ 0.15 and ≥ 0.2 missed between 7 and 31% of ≥ GG2 cancers and 3–20% of ≥ CPG3 tumours (Table 3).

**Table 3:** Diagnostic test statistics of the study cohort showing the accuracy and missed cancer rates for each *phi* threshold as a triage test pre-MRI and biopsy. PSAd thresholds are shown as a comparator though this metric is not usually available before an mpMRI. Detection rates for cancer are shown using definitions of ≥ Grade Group 2 (GG2) or ≥ Cambridge Prognostic Group 3 [CPG3]. (*percentage out of 258 cancers detected, ** percentage out of 176 cancers detected)

	**≥ GG2 cancer detection**					**≥ CPG3 cancer detection**			
	**Threshold**	**Sensitivity**	**Specificity**	**NPV**	% cancers missed*	**Sensitivity**	**Specificity**	**NPV**	% cancers missed**
***phi***	20	0.99	0.10	0.90	1.1	1.00	0.08	1.00	0
	25	0.96	0.25	0.87	4.2	0.99	0.22	0.99	0.05
	30	0.92	0.40	0.85	7.7	0.95	0.35	0.94	1.0
	35	0.87	0.55	0.83	12.8	0.93	0.49	0.93	7.3
**PSAd**	0.10	0.93	0.31	0.82	7.3	0.97	0.28	0.95	2.8
	0.15	0.81	0.57	0.77	18.6	0.90	0.53	0.92	9.6
	0.20	0.69	0.77	0.73	31.3	0.80	0.72	0.88	20.0

### Impact of using *phi* as a triaging test into an image-guided diagnostic pathway

We next compared different pathways to model referrals with and without use of *phi*. Table 4 summarises the key modelling results for each strategy, based on a hypothetical cohort of 1000 men referred for suspected prostate cancer. The base case model was an mpMRI and biopsy for all which detected every cancer but required more than half of men (53%) to undergo unnecessary biopsies (i.e. benign histology or ≤ GG2) (Table 4). Each alternative option resulted in an overall reduction in biopsy procedures, with the most impactful being a strategy to only biopsy men with positive mpMRI (M3–5) or to only biopsy men with a *phi* of ≥ 30 (23% and 25% reduction respectively). *phi* ≥ 30 also achieved this with a concomitant 25% reduction in mpMRI use by virtue of being an upfront triage test. We next assessed the impact on rates of detection of significant cancers. Using detection of ≥ GG2 as an endpoint, a strategy of mpMRI and biopsy only positive cases reduced unnecessary procedures by 35% but did miss 9% of tumours. In contrast, the *phi* ≥ 30 option to triage in referrals reduced unnecessary biopsies by 40% and missed 8% of ≥ GG2 cancers. The *phi* ≥ 25 option missed even fewer ≥ GG2 cancers (4%), but only reduced unnecessary biopsies by 25%. Using detection of ≥ CPG3 as an endpoint, both strategies of mpMRI and biopsy-positive lesions and using *phi* ≥ 30 led to similarly low missed cancer detection rates (5%); however, the *phi* ≥ 30 route required fewer unnecessary biopsies (31% vs 35%). Other pathways using PSAd (≥ 0.10 and 0.15) required many more overall biopsies (reductions of only 6–12%) and by definition all needed mpMRI (Table 4). These strategies did though miss many fewer ≥ GG2 tumours (1–2%) and no ≥ CPG3 disease (Table 4).

**Table 4:** Modelling results for a hypothetical cohort of 1000 patients referred for elevated PSA using different strategies for cancer detection. In brackets are projected reductions compared to the base model (* compared to MRI + biopsy all)

	**Pathway**					
	**MRI + biopsy all**	**MRI all + biopsy if M3–5**	**MRI all + biopsy if M3–5 or PSAd** ≥ **0.15**	**MRI all + biopsy if M3–5 or PSAd** ≥ **0.10**	**MRI + biopsy only if*phi*** ≥ **25**	**MRI + biopsy only if*phi*** ≥ **30**
No. of MRI scans (% lower*)	1000	1000 (0%)	1000 (0%)	1000 (0%)	850 (−15%)	750 (−25%)
No. of biopsies needed (% lower*)	1000	772 (−23%)	884 (−12%)	941 (−6%)	850 (−15%)	750 (−25%)
**Detection of ≥ GG2 cancers**						
Cancers identified (% lower*)	100%	91% (−9%)	98% (−2%)	99% (−1%)	96% (−4%)	92% (−8%)
Unnecessary biopsies (% lower*)	527	343 (−35%)	420 (−20%)	471 (−11%)	396 (−25%)	314 (−40%)
**Detection of ≥ CPG3 cancers**						
Cancers identified (% lower*)	100%	95% (−5%)	100% (0%)	100% (0%)	99% (−1%)	95% (−5%)
Unnecessary biopsies (% lower*)	677	466 (−31%)	562 (−17%)	618 (−9%)	529 (−21%)	442 (−35%)
**Cost analysis**						
Cost/pt. (% lower*)	£965	£796 (−18%)	£879 (−9%)	£921 (−5%)	£869 (−10%)	£774 (−20%)

### Cost modelling and decision curve analysis

**Fig. 2:**
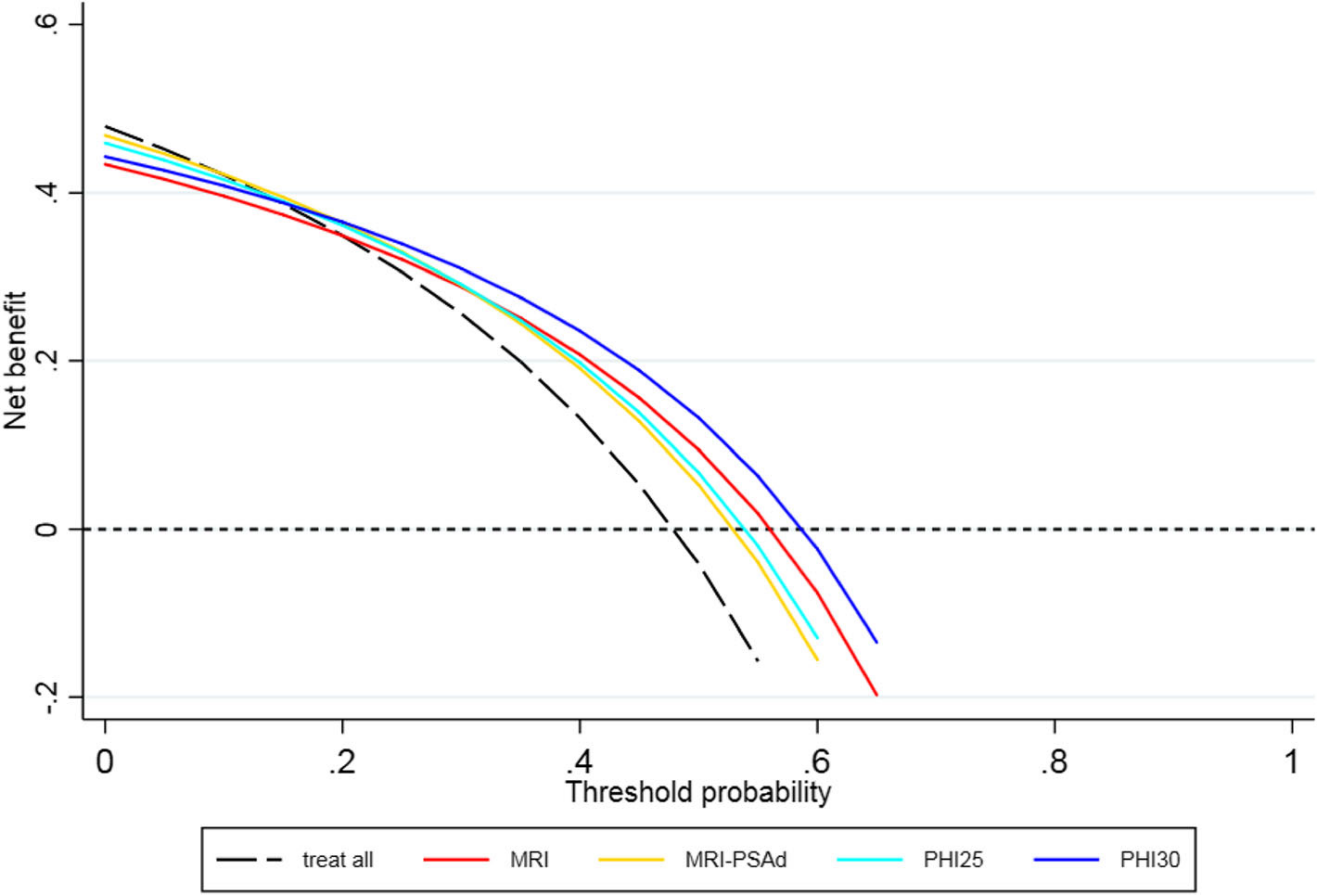
Decision curve analysis comparing the number of net benefits for detection of significant cancers for a range of risk threshold values and using different approaches (MRI-PSAd using a PSAd threshold of ≥ 0.15). MRI - magnetic resonance imaging, PSAd - PSA density, *phi* - Prostate Health Index

Table 4 includes the mean cost per investigated patient for each of the modelled diagnostic strategies. Compared to a cost of £965/person (mpMRI + biopsy) for all referred men, using a *phi* ≥ 30 threshold to restrict investigations reduced costs by 20% (£774/person). In contrast, a strategy of mpMRI + biopsy for only scan-positive cases was marginally costlier (£796/person). Strategies that involved using PSAd were also more expensive mainly due to the increased use of both mpMRI and biopsy (mean cost £879 and £921 for PSAd ≥ 0.15 and 0.10 respectively). Sensitivity analyses using higher rate or cost for sepsis following biopsy showed little impact on these comparisons (results not shown). Figure 2 shows the results of the decision curve analysis (incorporating the perceived harms of biopsying those without cancer) in terms of net clinical benefit resulting from different strategies based on ≥ GG2 tumour detection. At very low values of the risk threshold, biopsy all is the optimal strategy because these values represent a belief that there is little to no harm associated with an unnecessary biopsy. For risk thresholds 0.2–0.5 (the estimated range of uncertainty in clinical practice), the net benefit is highest under the *phi* ≥ 30 pathway. Since this is also the cheapest option (Table 4), this appears to be the optimal testing strategy representing a cost/cancer detected of between £2120 and £5860 (Additional File Table S3), depending on the risk threshold. However, for risk thresholds < 0.2 (i.e. where there is uncertainty to biopsy at cancer risk < 20%), the clinical net benefit is maximised by using more costly strategies. The optimal decision at these lower-risk thresholds therefore depends on the willingness to pay/cancer detected (Additional File Table S3). Hence for risk thresholds < 0.2, the cost for the *phi* ≥ 30 strategy is around £2000/cancer detected but more cancers (net) could be found using other strategies, at the cost of £4000 to £8000/additional cancer depending on the pathway.

## Discussion

In this paper, we report that use of the *phi* as a triage test could reduce both imaging and biopsies by a quarter while maintaining diagnostic efficiency using two definitions of clinically significant prostate cancers. We further demonstrate that introducing the *phi* is likely to be both the cheapest per referred patient and cheapest per net tumour detected.

The *phi* is one of many biomarkers reported in the last 10 years that have shown stronger predictive accuracy compared to PSA in detecting prostate cancer [20, 21]. Early studies compared these biomarkers head to head against mpMRI, but inevitably mpMRI proved superior as not only does it improve detection but also helps guide biopsies [28–30]. Subsequent studies have sought to combine biomarkers with mpMRI with all showing consistently better results than biomarkers alone but often in a retrospective setting. One notable prospective study combined the Stockholm3 test with mpMRI and showed the combination was more accurate than each individual test in detecting prostate cancer [14]. These studies, however, have generally not considered the incremental cost implications and therefore whether tests can be used sequentially [11, 31].

Recent work improving risk calculator performance using biomarkers and/or mpMRI have tended to use prostate biopsy as the end point [32, 33]. One exception is the work by Mannaerts et al. who retrospectively applied the Rotterdam Prostate Cancer risk calculator in 200 men and proposed that the calculator could have reduced mpMRI by 37% [34]. An updated calculator incorporating mpMRI was developed but in a recent prospective study did not improve prediction in biopsy naïve men but did do so for a re-biopsy population [35]. This suggests that simply modifying existing calculators with new parameters may need careful re-evaluation to test applicability. Particularly, if once “free” calculators start to incorporate costly biomarkers and imaging data points. In this regard, an advantage of our current study is its prospective application in a real-world setting and simultaneous cost analysis.

Our cost modelling suggests that a *phi*-based triage pathway may be less costly than other existing strategies. This is particularly relevant as mpMRI is now a mandatory pre-requisite step before prostate biopsy and hence an essential diagnostic cost [5]. Bi-parametric instead of multiparametric MRI may be cheaper but their comparative effectiveness remains debated [36, 37]. The cost of biomarkers also remains controversial with different studies showing varying results [38–40]. It is however notable that the *phi* test as costed by the manufacturer appears to be the most affordable amongst available biomarkers [19]. In this study, we found that it was both the cheapest per referred patient and the cheapest per tumour detected. Although other strategies (mpMRI and biopsy all or using PSAd) detected more prostate cancers, it came at a significantly higher cost as a result of having to undertake many more procedures.

This paper has many strengths. Key is the multicentre design in five different image-guided standard of care prostate diagnostic pathways. These are also limitations as there was no central quality assurance of biopsy method, histology and imaging. We also cannot account for decisions to not do biopsy if an mpMRI was negative as practice differed amongst sites and was evolving. Despite this, we were reassured to see that cancer detection rates and *phi* test performance were similar across centres. Detection rates were also comparable if not higher than many other published series which have used much more stringent trial parameters [41–43]. Our cost modelling was based on UK tariffs and extrapolation to other settings is dependent on individual tariffs in other countries. We note however that mpMRI-*phi* cost differentials are much greater in Europe and the USA. We did not compare performance of the *phi* with other biomarkers as these were not available to us. The Stockholm3 study showed very comparable results with a 10% risk threshold reducing mpMRI and biopsies by 40% and missing 8% of cancers [14]. A cost analysis was not published.

## Conclusion

We present here a first study reporting use of the *phi* test as a way of refining and reducing both mpMRI and biopsies in investigating suspected prostate cancer. Sequential use of the *phi* and then mpMRI ± biopsy may therefore be an efficient and effective way of identifying those men who will benefit most from investigation hence reducing cost and resource use in a rapidly growing disease demographic.

## Supplementary information

Additional file 1: **Figure S0.** Flow chart of recruitment and tests and final numbers analysed. **Figure S1.** ROC curve illustrating performance of PHI, PSA, PSAD and mpMRI in predicting cancer diagnosis of Cambridge Prognostic Group 3 [CPG3] in A. Whole cohort and B. mpMRI negative men (PI-RADS≤3). **Figure S2.** Decision tree pathway and percentages based on *phi*>30 pathway. **Table S1.** Profile of imaging and diagnostic method at each of the centres in the study. **Table S2.** Model parameters: base case values. Costs ascribed for each event. **Table S3.** Costs per net cancer detected, by risk threshold. It is assumed that at risk thresholds >0.5 (risk of cancer >50%), there would be clinical consensus that the perceived harms of biopsy are outweighed by the benefits of detecting and treating any cancer).

## Data Availability

All data is available by application to the study authors.
