# Supplementary material for: Clinical utility and cost modelling of the phi test to triage referrals into image-based diagnostic services for suspected prostate cancer: the PRIM (Phi to RefIne Mri) study
Source: BMC Med. 2020 Apr 17;18:95. doi: 10.1186/s12916-020-01548-3 (PMC7164355; doi:10.1186/s12916-020-01548-3)
Supplement: Supplementary file 1 — Figure S0. Flow chart of recruitment and tests and final numbers analysed. Figure S1. ROC curve illustrating performance of PHI, PSA, PSAD and mpMRI in predicting cancer diagnosis of Cambridge Prognostic Group 3 [CPG3] in A. Whole cohort and B. mpMRI negative men (PI-RADS≤3). Figure S2. Decision tree pathway and percentages based on phi>30 pathway. Table S1. Profile of imaging and diagnostic method at each of the centres in the study. Table S2. Model parameters: base case values. Costs ascribed for each event. Table S3. Costs per net cancer detected, by risk threshold. It is assumed that at risk thresholds >0.5 (risk of cancer >50%), there would be clinical consensus that the perceived harms of biopsy are outweighed by the benefits of detecting and treating any cancer). [file 12916_2020_1548_MOESM1_ESM.docx]

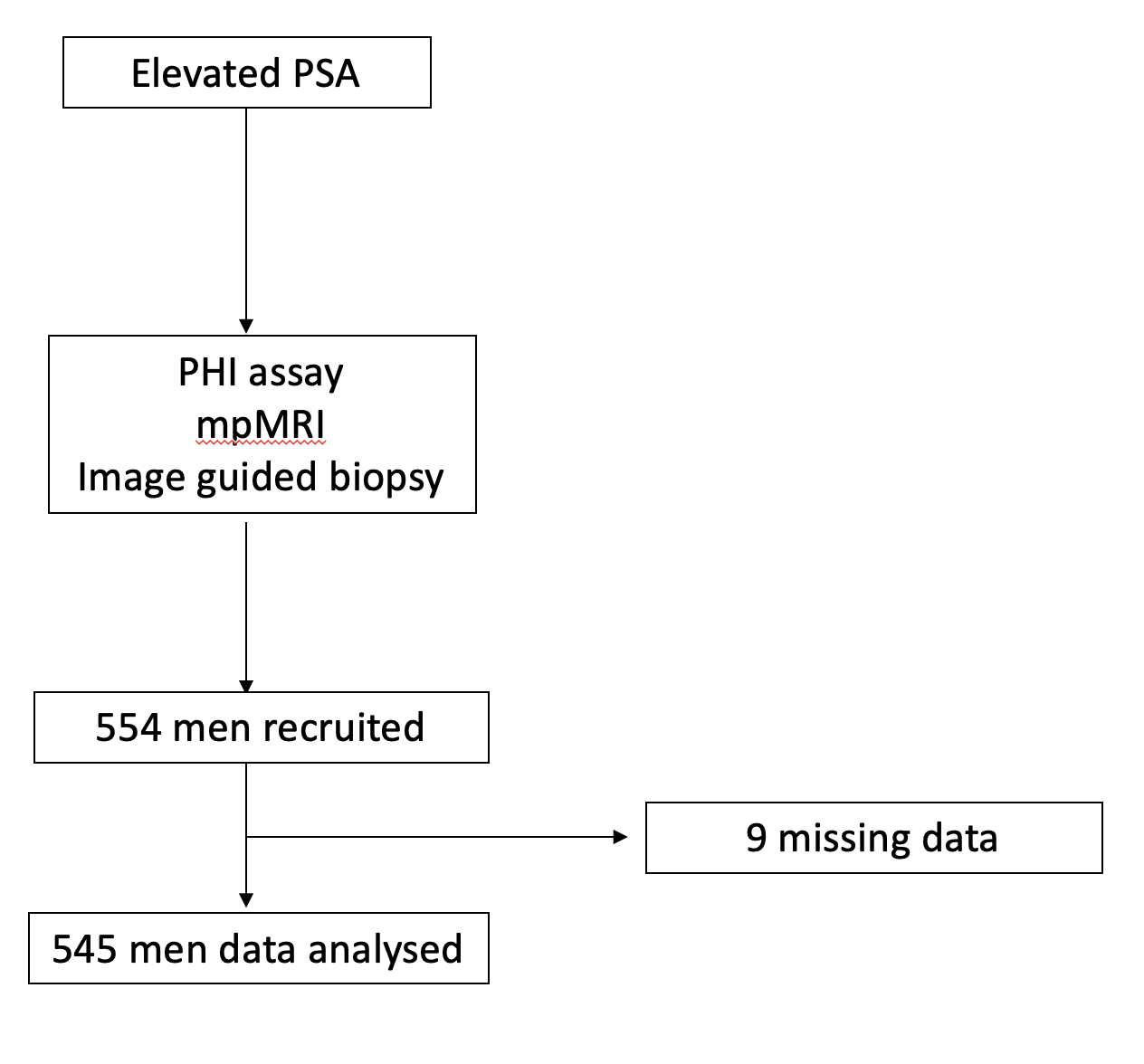


**Additional File Figure S0** - Flow chart of recruitment and tests and final numbers analysed

**A**


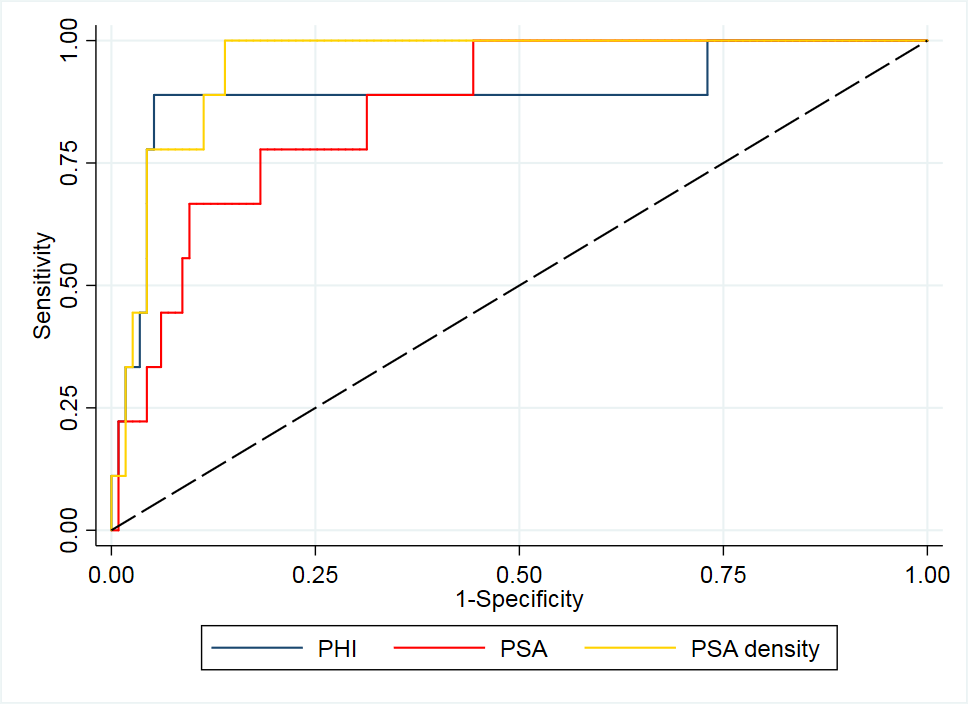


**B**

**Additional File Figure S1** – ROC curve illustrating performance of PHI, PSA, PSAD and mpMRI in predicting cancer diagnosis of Cambridge Prognostic Group 3 [CPG3] in **A.** Whole cohort and **B**. mpMRI negative men (PIRADS≤3).

**Additional File Figure S2** - Decision tree pathway and percentages based on *phi*>30 pathway

| **Centre** | **MRI magnet** | **PiRADS 4-5 positive rate** | **Image guidance** | **Biopsy method** | **Cancer detection rate**  **(≥ GG2) *** | **Cancer detection**  **rate**  **(≥ CPG3) **** |
| --- | --- | --- | --- | --- | --- | --- |
| **1** | 1.5T | 45% | Cognitive | TRUS (77%) & TP (23%) | 60% | 28% |
| **2** | 1.5T | 51% | Cognitive TRUS/TP fusion | TRUS (92%) & TP (8%) | 41% | 33% |
| **3** | 1.5T | 76% | Cognitive | TRUS (84%) & TP (16%) | 47% | 33% |
| **4** | 3T/1.5T | 58% | Fusion | TRUS (67%) & TP (33%) | 47% | 34% |
| **5** | 1.5T | 63% | Cognitive | TP (100%) | 46% | 25% |

**Additional File Table S1** - Profile of imaging and diagnostic method at each of the centres in the study. Detection rates for cancer were similar across all centre using definitions of ≥ Grade Group 2 (GG2) *p=0.47 or ≥ Cambridge Prognostic Group 3 [CPG3] **p= 0.56. TRUS- transrectal ultrasound guided biopsies. TP- transperineal prostate biopsies. The median number of cores taken was 16 for systematic biopsies and 2 for targets (if positive on an mpMRI). Cognitive and fusion biopsies have been shown to have equivalent cancer detection rates (43).

| **Event** | **Value** | **Source** |
| --- | --- | --- |
| PHI test | £50 | Beckman Coulter |
| mpMRI scan (including out-patient appointment) | £223 | NHS tariff (26)* |
| Biopsy** | £650 | NHS tariff (26)** |
| Sepsis treatment costs (per case) | £4260 | Batura *et al* (27) |
| Percentage of biopsies proceeding to sepsis | 2.15% | Batura *et al* (27) |

* code RD02A

** calculated as a weighted average of TRUS biopsy (£329 -code LB76Z, 58% of biopsies) and transperineal (£1093 -code LB77Z, 42% of biopsies)

**Additional File Table S2** – **Model parameters: base case values.** Costs ascribed for each event

| **Risk threshold** | **Cost per net cancer detected**  **under PHI**≥**30 pathway** | **Additional cost per additional net cancer detected under MRI/PSAd>0.15 pathway*** | **Additional cost per additional net cancer detected under biopsy all pathway*** |
| --- | --- | --- | --- |
| **0.5** | £5860 | NA | NA |
| **0.4** | £3280 | NA | NA |
| **0.3** | £2500 | NA | NA |
| **0.2** | £2120 | NA | NA |
| **0.1** | £1890 | £7690- | NA |
| **0.05** | £1810 | £5300 | £7600 |
| **0** | £1750 | £4150 | £5280 |

**Additional File Table S3 :** Costs per net cancer detected, by risk threshold. It is assumed that at risk thresholds >0.5 (risk of cancer >50%), there would be clinical consensus that the perceived harms of biopsy are outweighed by the benefits of detecting and treating any cancer). * compared to PHI≥30 (the least costly and highest clinical net benefit at RT ≥0.2); NA = not applicable (pathway is both more expensive and less clinically effective than PHI≥30 at this risk threshold)
